# The inter-link of ageing, cancer and immunity: findings from real-world retrospective study

**DOI:** 10.1186/s12979-023-00399-9

**Published:** 2023-12-15

**Authors:** Xiaomin Fu, Peng Qin, Fanghui Li, Huifang Zhu, Hongqin You, Yong Zhang, Benling Xu, Tiepeng Li, Fang Zhang, Lu Han, Lingdi Zhao, Baozhen Ma, Zibing Wang, Quanli Gao

**Affiliations:** 1 Department of Immunotherapy, Affiliated Cancer Hospital of Zhengzhou University and Henan Cancer Hospital, Zhengzhou, 450003 China; 2 GMP Laboratory of Immunotherapy, Affiliated Cancer Hospital of Zhengzhou University and Henan Cancer Hospital, Zhengzhou, 450003 China

**Keywords:** Ageing, Cancer, Immunity, PD-1, T cell

## Abstract

**Background:**

Although the concept of declined immune function associated with cancer has been accepted extensively, real-world clinical studies focusing on analysis of the peripheral blood immune changes underlying ageing, immunity and cancer are scarce.

**Methods:**

In this case-control study, we retrospectively analysed 1375 cancer patients and enrolled 275 age and gender matched healthy individuals. Flow cytometry was conducted to assess the immune changes. Further analysis was examined by SPSS 17.0 and GraphPad Prism 9 software.

**Results:**

Cancer patients showed obviously decreased CD3^+^ T, CD3^+^CD4^+^ Th, CD3^+^CD8^+^ CTL, CD19^+^ B, CD16^+^CD56^+^ NK cell counts and lower percentage of PD-1 (programmed cell death protein-1, PD-1) positive cells than healthy control (*P* < 0.0001). For cancer patients, the reference range of circulating percentage of PD-1^+^CD45^+^ cells, PD-1^+^CD3^+^ T cells, PD-1^+^CD3^+^CD4^+^ Th cells and PD-1^+^CD3^+^CD8^+^ CTL (Cytotoxic T Lymphocyte, CTL) were 11.2% (95% CI 10.8%-11.6%), 15.5% (95% CI 14.7%-16.0%), 15.4% (95% CI 14.9%-16.0%) and 14.5% (95% CI 14.0%-15.5%), respectively. Moreover, the reduction of CD3^+^ T, CD3^+^CD4^+^ Th, CD3^+^CD8^+^ CTL, CD19^+^ B cell counts accompanied with age and stage advancing (*P* < 0.05). CD16^+^CD56^+^ NK cells decreased with stage, but elevated in aged and male cancer patients (*P* < 0.05). Additionally, the percentage of PD-1 positive cells varied across cancer types, raised with age and stage. Head and neck, pancreatic, gynaecological and lung demonstrated a higher level of the percentage of PD-1 positive cells than melanoma, prostate, and breast cancer (*P* < 0.05).

**Conclusions:**

This study provides the reference range of the percentage of PD-1 positive cells on peripheral blood, confirms the decreased immune cells and a series of immune changes accompanying with cancer, expands our real world evidence to better understand the interactions of ageing, cancer and immunity. Moreover, the circulating percentage of PD-1 positive cells shows similar tumor type distribution with tumor mutational burden (TMB), supports that it maybe a potential predictive biomarker for immune checkpoint inhibitor therapy.

**Supplementary Information:**

The online version contains supplementary material available at 10.1186/s12979-023-00399-9.

## Background

Ageing plays an important role in the composition and function of the immune system, which leading to an increased susceptibility to cancer [1]. Most of cancers are predominantly diagnosed over 60 years old [2]. For both sexes globally, cancer has become a leading cause of death and serious health burden [3]. In China, lung, gastric, liver, colorectal, esophagus and bladder are the most common cancer types for males. As for females, cancers are more common in breast, lung, colorectal, thyroid, gastric and cervix [4].

In human beings, CD45 expressed on all leukocytes surface and regulated T and B cell signalling [5]. T cells can be divided into two major subsets, CD4^+^ and CD8^+^, based on the expression of surface molecules [6]. In recent years, CD8^+^ T cells have gained more attention for their cytolytic activities by releasing perforin and granzymes in anti-cancer immunity [7, 8]. B cells play a major role in chronic inflammatory diseases by antibody secretion, antigen presentation, and T cell regulation [9]. Decreased B cells can impair antibody responses, deeply influence the chronic inflammatory disease development and its progression [10]. NK cells reside in different tissues, eliminate target cells through cell contacted and cytokine secreted patterns, act as a connection of innate and adaptive immunity [11–13].

Most published studies investigating age-related changes in immune function are in murine models or healthy populations. Correspondingly, decreased T and B cell numbers have been reported accompanied with age advancing and contributed to immune dysfunctions [14, 15]. Besides, ageing-related reduction in CD8^+^ T cell number occurs in the lymph nodes, along with an increased ratio of CD4^+^/CD8^+^ T cells [16]. Studies reported increased or maintained NK cells in the elderly population, although proliferation rates and migration function appear to be decreased [17, 18].

Cancer seems to alter both the innate and adaptive immunity of individuals [19]. T cell-based adaptive immunity is pivotal to maintain immune homeostasis, purge cancer cells and inhibit tumor progression [20]. Programmed cell death protein-1 (PD-1) is a co-inhibitory receptor to suppress T cell over-activation and has becoming an important cancer immunotherapy target [21]. The higher percentage of PD-1^+^CD8^+^ T cells in tumor microenvironment (TME) exhibited predictive value to the efficacy of PD-1 blockade therapy [22]. Due to the difficult work to obtain TME phenotypes dynamically, it is more feasible to observe the immune status by using peripheral blood [23]. However, the profiles of the tumor associated changes in circulating PD-1 positive cells remain insufficiently studied.

Accumulated evidence supported that the baseline peripheral immune phenotype was related to the prognosis of anti-PD-1 immunotherapy. Reports suggested that higher level of PD-1^+^CD3^+^CD4^+^ Th cells correlated with better survival in non-small cell lung cancer (NSCLC) patients [24, 25]. Our previous study indicated that the higher level of baseline CD16^+^CD56^+^ NK cell counts and the ratio of CD4^+^/CD8^+^ T cells in peripheral blood could predict longer progression free survival (PFS) and better response in NSCLC patients treated with anti-PD-1 based therapy [26]. Beyond T and NK cells, baseline lower B cell numbers and its percentage associated with a superior prognosis of immune checkpoint treatment have also been certified by our and other teams [27, 28]. To date, rare studies focus on systemically analysing peripheral blood immunophenotypes in cancer patients by flow cytometry.

In this study, we aimed to compare the immune differences between cancer patients and healthy individuals, clarify the immunological characteristics in cancer patients and investigate the value of circulating percentage of PD-1 positive cells and its impact factors.

## Results

### Immune-phenotype variation between cancer patients and healthy populations

The absolute number of immune cells in peripheral blood was compared and outlined in supplementary Table 1. Considering the healthy population recruited in our study was limited and in accordance with the human reference range. We used the reference range of immune cell counts from BD Bioscience Company as healthy control. An obviously decreased absolute numbers were observed in cancer patients, including CD45^+^, CD3^+^ T, CD3^+^CD4^+^ Th, CD3^+^CD8^+^ CTL, CD16^+^CD56^+^ NK and CD19^+^ B cells (*P* < 0.0001).

**Fig. 1:**
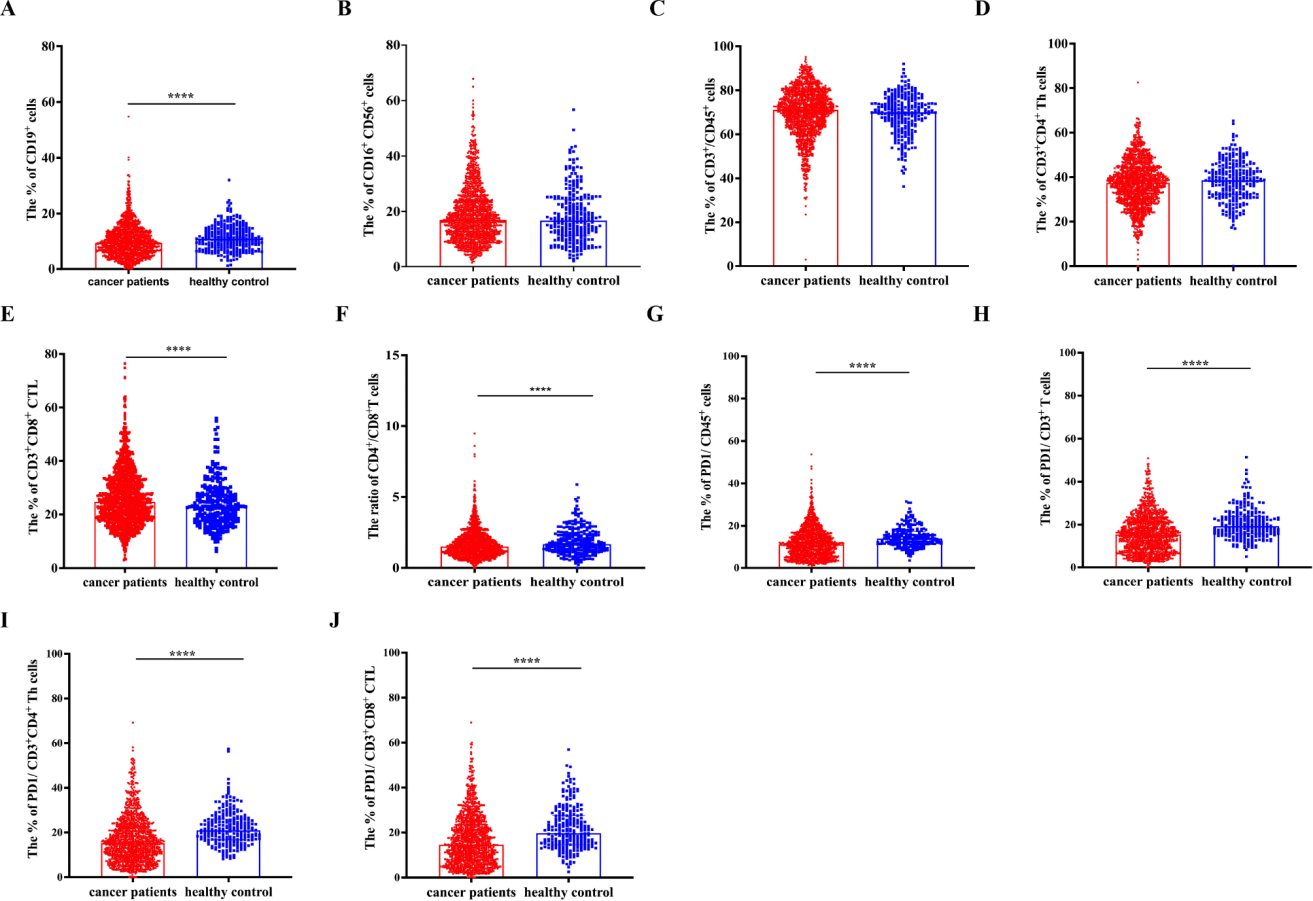
Immune phenotype variation between cancer patients and healthy populations. The peripheral blood mononuclear cells (PBMC) of cancer patients (*n* = 1375) and healthy control (*n* = 275) were detected by flow cytometry. **(A-E)** The percentage of CD19^+^ B cells, CD16^+^ CD56^+^ NK cells, CD3^+^CD45^+^ cells, CD3^+^CD4^+^ Th cells, and CD3^+^CD8^+^ CTL in the two groups. **(F)** The ratio of CD4^+^/CD8^+^ T cells. **(G-J)** The percentage of PD-1^+^CD45^+^ cells, PD-1^+^CD3^+^ T cells, PD-1^+^ CD3^+^CD4^+^ Th cells, and PD-1^+^CD3^+^CD8^+^ CTL. Data were shown as mean ± SEM. Statistic differences between the two groups were detected by unpaired t-tests and were indicated as follows: *p < 0.05, **p < 0.01, ***p < 0.001, and ****p < 0.0001

The percentage of immune cells was investigated and shown in Fig. 1. The percentage of CD19^+^ B cells was higher in cancer patients compared with healthy control (median: 10.95% vs. 9.5%, *p* < 0.0001, Fig. 1A). There was no difference in the percentage of CD16^+^CD56^+^ NK cells between two groups (Fig. 1B). As for the T cell subsets, no difference was found in the percentage of CD3^+^CD45^+^ cells and CD3^+^CD4^+^ Th cells (Fig. 1C and D). Of note, the cancer group had higher percentage of CD3^+^CD8^+^ CTL (median: 24.7% vs. 22.8%, *p* = 0.0003, Fig. 1E) and lower ratio of CD4^+^/CD8^+^ T cells than the healthy control (median: 1.496 vs.1.67, *p* = 0.0007, Fig. 1F).

The percentage of PD-1^+^CD45^+^ cells, PD-1^+^CD3^+^ T cells, PD-1^+^CD3^+^CD4^+^ Th cells and PD-1^+^CD3^+^CD8^+^ CTL in both groups were analysed. For cancer patients, the established percentage reference range of PD-1^+^CD45^+^ cells, PD-1^+^CD3^+^ T cells, PD-1^+^CD3^+^CD4^+^ Th cells and PD-1^+^CD3^+^CD8^+^ CTL were 11.2% (95% CI 10.8%-11.6%), 15.5% (95% CI 14.7%-16.0%), 15.4% (95% CI 14.9%-16.0%) and 14.5% (95% CI 14.0%-15.5%), respectively. All the percentage of PD-1^+^ cells in cancer patients were significantly lower than healthy group (*p* < 0.0001, Fig. 1G-J).

### Age and gender related immune changes in cancer patients

**Fig. 2:**
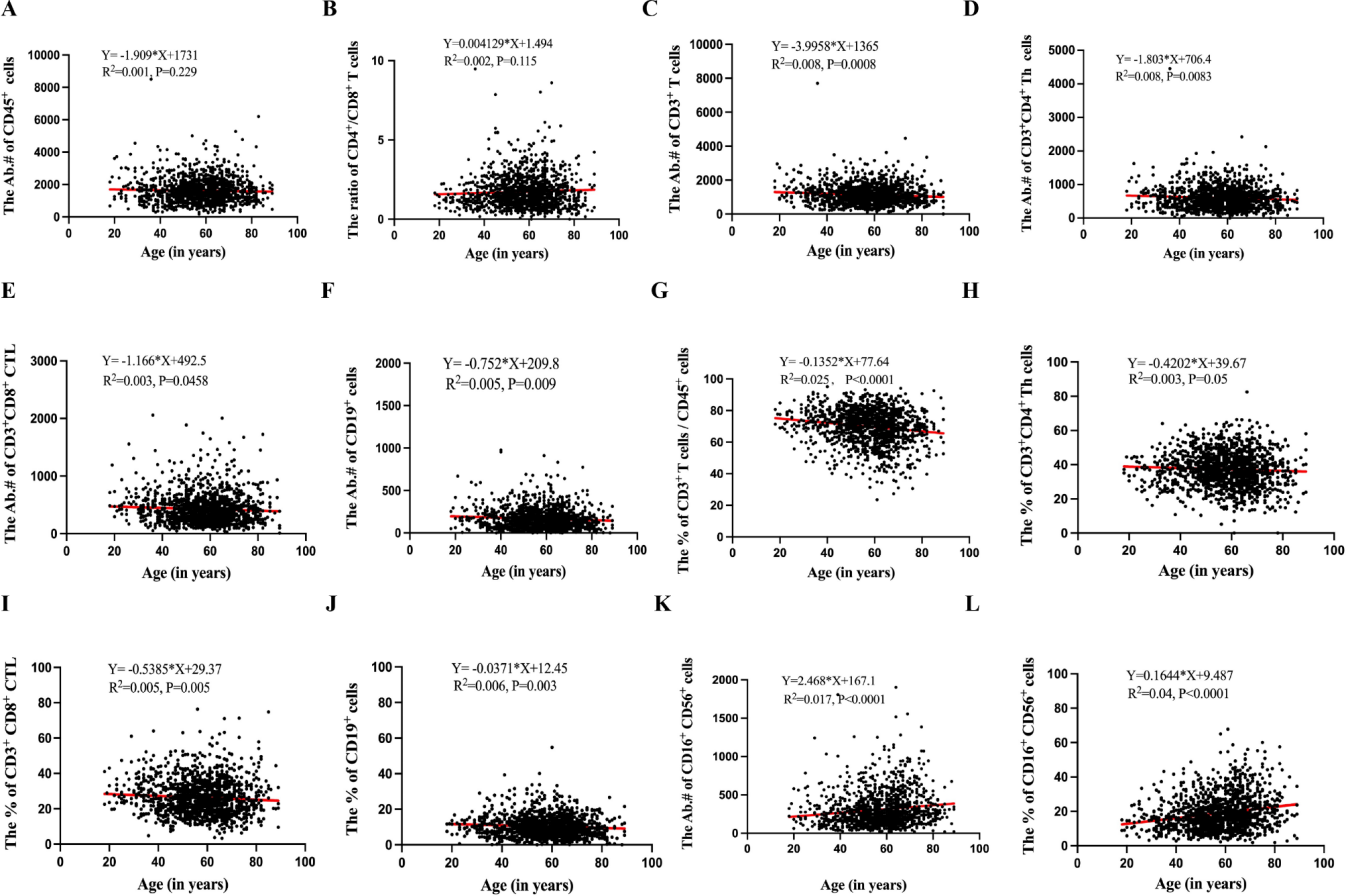
Age-related immune shifts in cancer patients. **(A)** The correlation between the absolute number of CD45^+^ cells and age. **(B)** The correlation between the ratio of CD4^+^/CD8^+^ T cells and age. **(C-F)** The correlation between the absolute number of CD3^+^ T cells, CD3^+^CD4^+^ Th cells, CD3^+^CD8^+^ CTL, CD19^+^ B cells and age. **(G-J)** The correlation between the percentage of CD3^+^CD45^+^ cells, CD3^+^CD4^+^ Th cells, CD3^+^CD8^+^ CTL, CD19^+^ B cells and age. **(K)** The correlation between the absolute number of CD16^+^CD56^+^ NK and age. **(L)** The correlation between the percentage of CD16^+^CD56^+^ NK cells and age. The Pearson correlation coefficient was computed to evaluate correlations between two parameters

Cancer patients were divided into three subgroups according to age (≤ 35, 36–60, > 60 years old). Circulating CD45^+^ (R^2^ = 0.001, *P* = 0.229, Fig. 2A) cell numbers and the ratio of CD4^+^/CD8^+^ T cells (R^2^ = 0.002, *P* = 0.115, Fig. 2B) were stable during the whole life span. However, cell counts of CD3^+^ T (R^2^ = 0.008, *P* = 0.0008, Fig. 2C), CD3^+^CD4^+^ Th (R^2^ = 0.008, *P* = 0.0083, Fig. 2D), CD3^+^CD8^+^ CTL (R^2^ = 0.003, *P* = 0.0458, Fig. 2E) and CD19^+^ B (R^2^ = 0.005, *P* = 0.009, Fig. 2F) reduced with age progression. In addition, the percentage of CD3^+^CD45^+^ cells (R^2^ = 0.025, *P* < 0.0001, Fig. 2G), CD3^+^CD4^+^ Th cells (R^2^ = 0.003, *P* = 0.050, Fig. 2H), CD3^+^CD8^+^ CTL (R^2^ = 0.005, *P* = 0.0050, Fig. 2I) and CD19^+^ B cells (R^2^ = 0.006, *P* = 0.003, Fig. 2J) also decreased with advancing age. By contrast, both CD16^+^CD56^+^ NK cell count (R^2^ = 0.017, *P* < 0.0001, Fig. 2K) and its percentage (R^2^ = 0.04, *P* < 0.0001, Fig. 2L) increased with age.

**Fig. 3:**
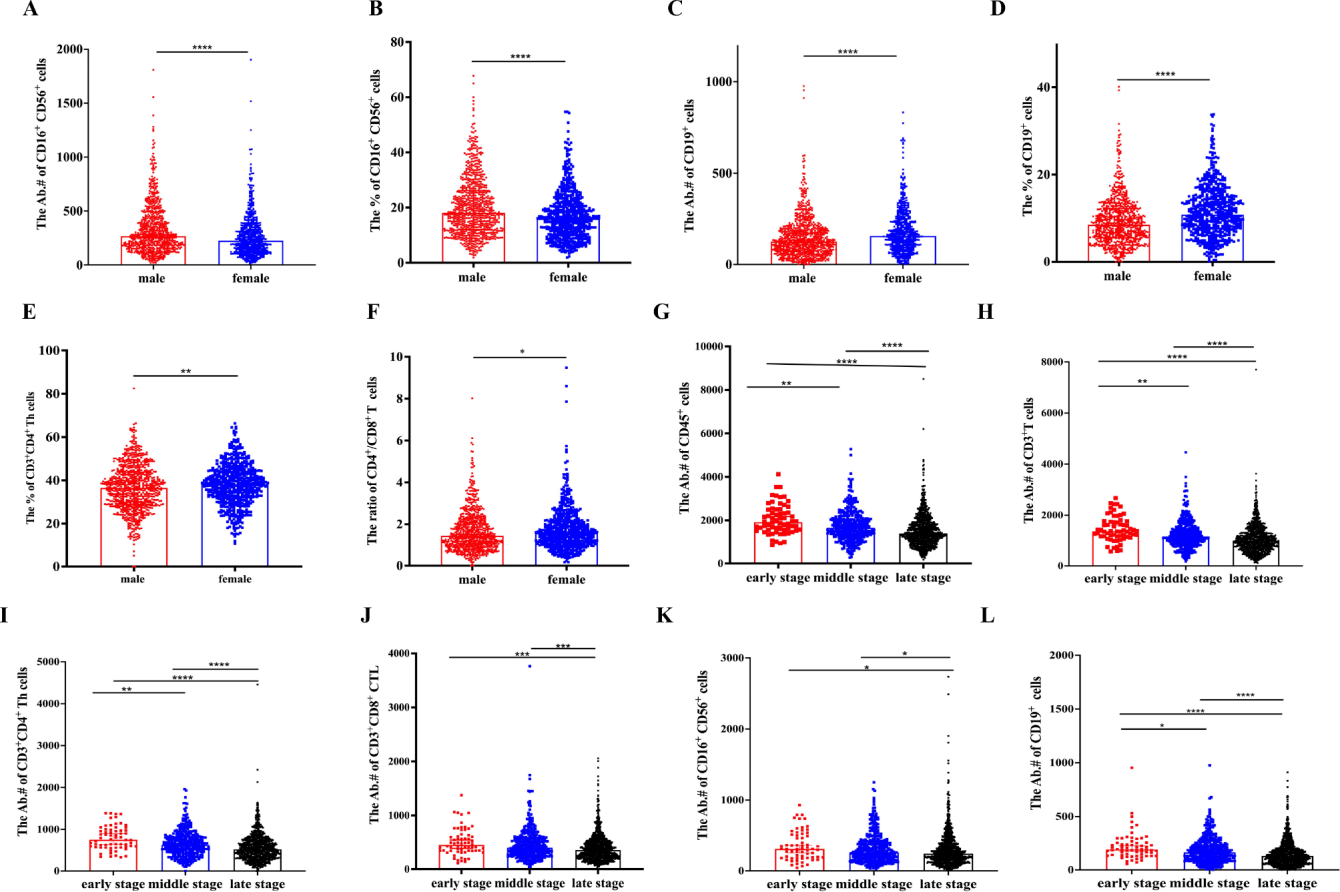
Gender and stage could influence the immune cells in cancer patients. **(A-B)** The absolute number and percentage of CD16^+^CD56^+^ NK cells in male (*n* = 770) and female (*n* = 605) cancer patients. **(C-D)** The absolute number and percentage of CD19^+^ B cells in male and female patients. **(E-F)** The percentage of CD3^+^CD4^+^ Th cells and the ratio of CD4^+^/CD8^+^ T cells in two groups. **(G-L)** The absolute number of CD45^+^ cells, CD3^+^ T cells, CD3^+^CD4^+^ Th cells, CD3^+^CD8^+^ CTL, CD16^+^ CD56^+^ NK cells, and CD19^+^ B cells among early (*n* = 64), middle (*n* = 386) and late stage (*n* = 925) of cancer patients. Data were shown as mean ± SEM. Statistic differences between two groups were detected by unpaired t-tests. As for data containing more than two groups, they were compared by one-way ANOVA test followed by the Turkey test. *P* values were indicated as follows: *p < 0.05, **p < 0.01, ***p < 0.001, and ****p < 0.0001

Compared to female cancer patients, the male had more cell counts and higher percentage of CD16^+^CD56^+^ NK cells (*P* < 0.0001, Fig. 3A and B), but fewer cell counts and lower percentage of CD19^+^ B cells (*P* < 0.0001, Fig. 3C and D). Moreover, the percentage of CD3^+^CD4^+^ Th cells and the ratio of CD4^+^/CD8^+^ T cells were found lower in males than females (*P* < 0.01, Fig. 3E, *P* < 0.05, Fig. 3F). Gender did not seem to influence the cell counts of CD45^+^, CD3^+^ T, CD3^+^CD4^+^ Th, CD3^+^CD8^+^ CTL, the percentage of CD3^+^CD45^+^ cells and CD3^+^CD8^+^ CTL. (*P* > 0.05, Supplementary Fig. 2A-2 F).

### Tumor stage influence on immune cells, and tumor distribution in age and gender subgroups

The tumor stage was another important factor to affect immune function. Cell counts of CD45^+^, CD3^+^ T, CD3^+^CD4^+^ Th, CD3^+^CD8^+^ CTL, CD16^+^CD56^+^ NK and CD19^+^ B demonstrated a decreasing trend as the disease entered its advanced stage (*P* < 0.05, Fig. 3G-L). No significant difference were noted in the percentage of CD3^+^CD45^+^ cells, CD16^+^CD56^+^ NK cells, CD19^+^ B cells, CD3^+^CD4^+^ Th cells, CD3^+^CD8^+^ CTL and the ratio of CD4^+^/CD8^+^ T cells amongst the tumor stage (Supplementary Fig. 3). However, cell counts of CD45^+^, CD3^+^ T, CD3^+^CD4^+^ Th, CD3^+^CD8^+^ CTL, CD19^+^ B and CD16^+^CD56^+^ NK were not affected by tumor type (*P* > 0.05, Supplementary Fig. 4).

We observed the distribution of major 14 cancer types in age and gender subgroups. 637 (48.4%) and 599 (45.5%) cases were diagnosed in middle and elder subgroup, respectively. Further analysis found that age influenced cancer distribution only identified in bone and soft tissue carcinoma in our study (*P* < 0.05, Supplementary Table 2). The gender bias of cancer incidence was obviously significant in our data. Exclusive of 160 (12.0%) cases were breast or gynaecological patients, the proportion of male patients reached nearly 64% (Supplementary Table 2).

### The percentage of PD-1 positive cell characteristics and their discrepancies in cancer patients

**Fig. 4:**
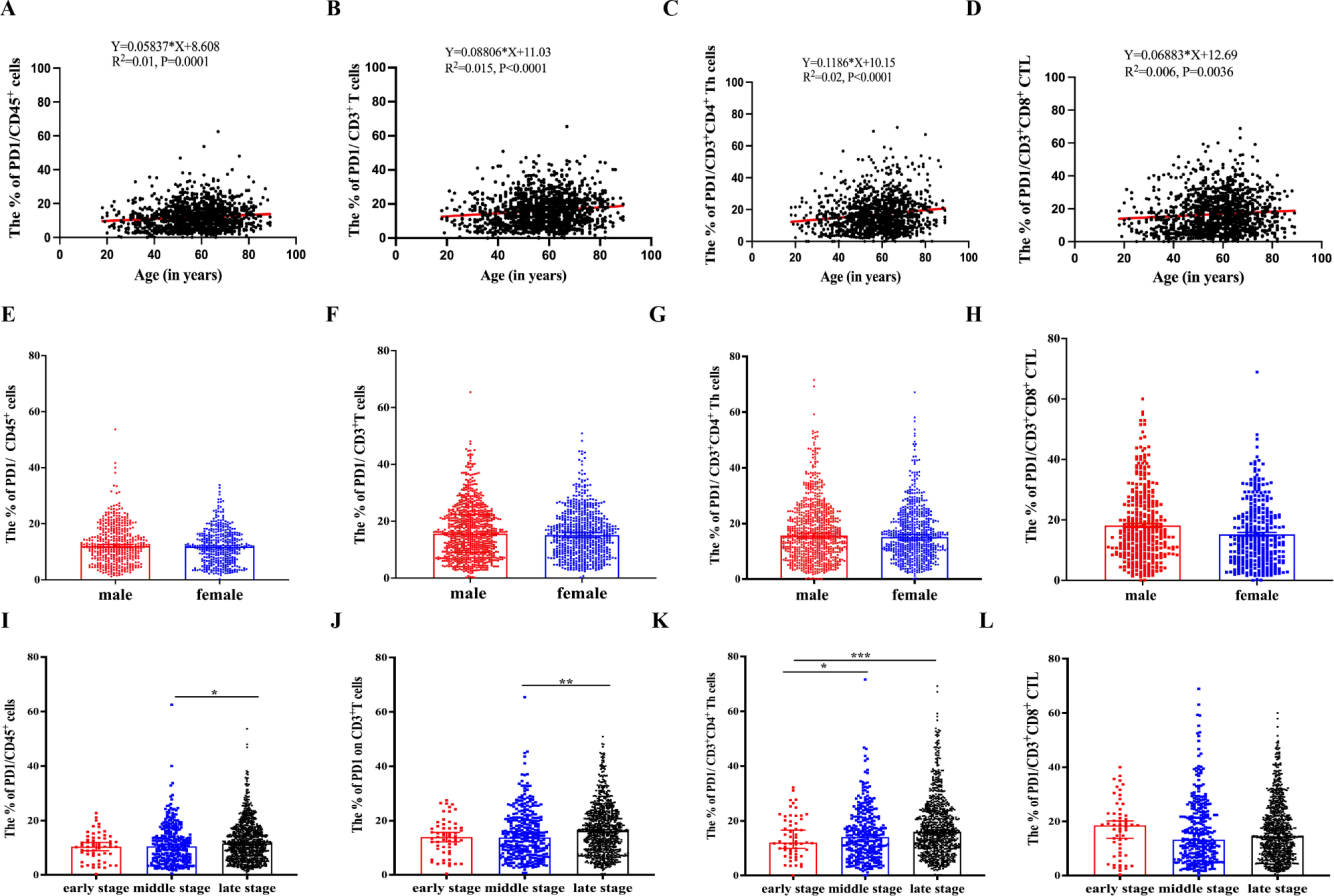
The PD-1^+^ T cells and its variation in cancer patients. **(A-D)** The correlation between the percentage of PD-1^+^CD45^+^ cells, PD-1^+^CD3^+^ T cells, PD-1^+^CD3^+^CD4^+^ Th cells, PD-1^+^CD3^+^CD8^+^ CTL and age in cancer patients. The Pearson correlation coefficient was computed to evaluate correlations between two parameters. **(E-H)** The percentage of PD-1^+^CD45^+^ cells, PD-1^+^CD3^+^ T cells, PD-1^+^CD3^+^CD4^+^ Th cells, and PD-1^+^CD3^+^CD8^+^ CTL in male (*n* = 770) and female (*n* = 605) cancer patients. **(I-L)** The percentage of PD-1^+^CD45^+^ cells, PD-1^+^CD3^+^ T cells, PD-1^+^CD3^+^CD4^+^ Th cells, and PD-1^+^CD3^+^CD8^+^ CTL among early, middle, and late stage. Data were shown as mean ± SEM. Statistic differences between two groups were detected by unpaired t-tests. As for data containing more than two groups, they were compared by one-way ANOVA test followed by the Turkey test. *P* values were indicated as follows: *p < 0.05, **p < 0.01, ***p < 0.001, and ****p < 0.0001

Overall, The percentage of PD-1^+^CD45^+^ cells (R^2^ = 0.01, *P* = 0.0001, Fig. 4A), PD-1^+^CD3^+^ T cells (R^2^ = 0.015, *P* < 0.0001, Fig. 4B), PD-1^+^CD4^+^ Th cells (R^2^ = 0.02, *P* < 0.0001, Fig. 4C) and PD-1^+^CD3^+^CD8^+^ CTL (R^2^ = 0.006, *P* = 0.0036, Fig. 4D) showed an increase trend with age in both cancer patients and healthy individuals (Supplementary Fig. 5).

Gender did not affect the percentage of PD-1^+^CD45^+^ cells, PD-1^+^CD3^+^ T cells, PD-1^+^CD3^+^CD4^+^ Th cells and PD-1^+^CD3^+^CD8^+^ CTL (*P* > 0.05, Fig. 4E-H). With cancer moving to advanced stage, the percentage of PD-1^+^CD45^+^ cells, PD-1^+^CD3^+^ T cells, PD-1^+^CD4^+^ Th cells showed an increased trend (*P* < 0.05, Fig. 4I-K). But, the similar stage-related trend was not found in PD-1^+^CD3^+^CD8^+^ CTL (*P* > 0.05, Fig. 4L).

**Fig. 5:**
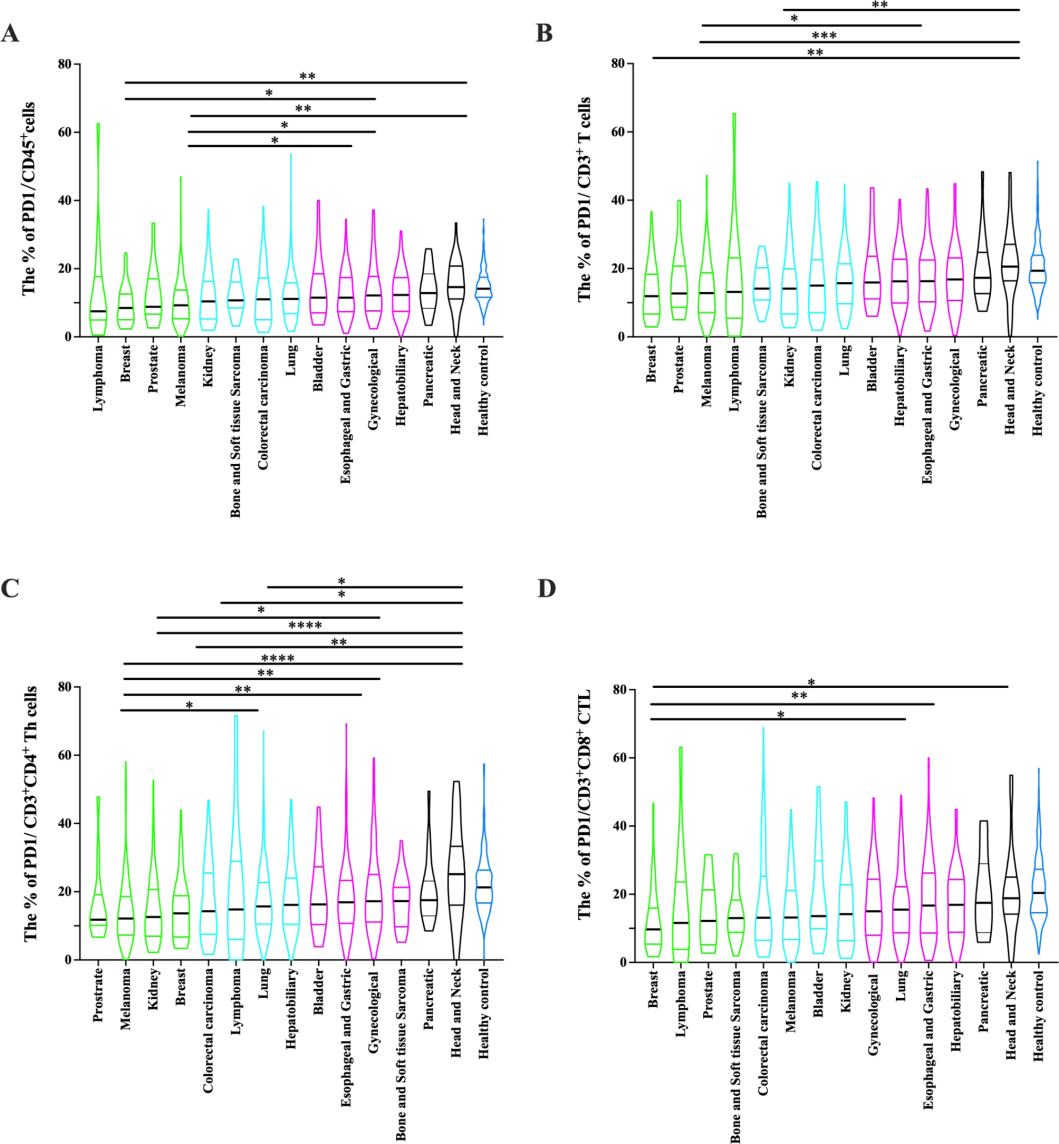
Comparison of the percentage of PD-1 positive cells among different cancer types. **(A-D)** The percentage of PD-1^+^CD45^+^ cells, PD-1^+^CD3^+^ T cells, PD-1^+^CD3^+^CD4^+^ Th cells, PD-1^+^CD3^+^CD8^+^ CTL in different cancer types including lung cancer (*n* = 307), esophagus and gastric cancer (*n* = 212), melanoma (*n* = 166), colorectal carcinoma (*n* = 129), kidney cancer (*n* = 121), gynecological cancer (cervical, ovarian and uterine cancer, *n* = 94), hepatobiliary cancer (*n* = 84), breast cancer (*n* = 66), head and neck cancer (*n* = 36), bone and soft tissue sarcoma (*n* = 35), bladder cancer (*n* = 21), pancreatic cancer (*n* = 18), lymphoma (*n* = 14), prostate cancer (*n* = 13). Data were shown as mean ± SEM and compared by one-way ANOVA test followed by the Turkey test. *P* values were indicated as follows: *p < 0.05, **p < 0.01, ***p < 0.001, and ****p < 0.0001

Tumor type did not influence the cell counts of CD45^+^, CD3^+^ T, CD3^+^CD4^+^ Th, CD3^+^CD8^+^ CTL, CD19^+^ B and CD16^+^CD56^+^ NK (*P* > 0.05, Supplementary Fig. 4). However, further analysis showed that the percentage of PD-1^+^ CD45^+^ cells, PD-1^+^CD3^+^ T cells, PD-1^+^CD3^+^CD4^+^ Th cells, PD-1^+^CD3^+^CD8^+^ CTL varied among major cancer types. Head and neck, pancreatic, gynaecological, esophagus and gastric cancer exhibited higher level of the percentage of PD-1 positive cells than melanoma, prostate, and breast cancer (*P* < 0.05, Fig. 5).

## Discussion

Rapid ageing has a severe disruption on the composition and function of the immune system, leading to an increased risk of cancer [29]. In particular, ageing T cell population and progressive loss of cellular function play pivotal role to eliminate abnormal cells, which can lead to the development of cancer [30].

The ageing immune system is featured by the shift of immune cell production, death, and differentiation. T and B lymphocytes are more feasible to be affected by the process of ageing [31–33]. The number of naive T cells decreased and the antigen-experienced T cells elevated with age, which increased the frequencies of terminal effector senescent or exhausted T cells [31]. These ageing influenced T cells reduced tumor killing capabilities and promoted tumor cell evasion from immune system [34].

In addition, tumor also induces T cell exhausion and sustains a suppressive tumor microenvironment [35]. Several studies indicate that T cell senescence occurring in patients with chronic viral infections, and in tumor-infiltrating lymphocytes (TILs) of various cancer types [35].

In this study, we investigated a case-control study in 1375 pan-cancer patients and gender and age matched healthy population, demonstrating a series of immune changes accompanying with cancer.

## Immune capacity decreases with cancer

Early studies pointed out that ageing related changes in the bone marrow showed impaired potency of lymphocytes generation, self-renewal and differentiation [32]. Besides, dysfunctional thymus co-contributed to the new T cell generation and replenishment [32]. Correspondingly, the production of neutrophils and monocytes was enlarged, whereas the generation of B and T lymphocytes was sharply diminished [36].

In line with previous studies, our results displayed declined cell counts of CD45^+^, CD3^+^ T, CD3^+^CD4^+^ Th, CD3^+^CD8^+^ CTL, CD16^+^CD56^+^ NK and CD19^+^ B in cancer patients. Lower immune cells contributed to the malfunction of the humoral and cellular immune systems. As a result, chronic inflammatory stimulation and immune dysfunction worked together to assist normal cells in moving toward malignancy, escaping immune surveillance, eventually growing to enlarged tumor tissues. For the T cell subsets, the cancer group had higher percentage of CD3^+^CD8^+^ CTL than healthy control. Etiologic factors associated with viral infections including human papillomavirus (HPV), Epstein-Barr virus (EBV), Hepatitis B virus (HBV) and so on, maybe the possible reason leading to the immune activation in cancer patients [10, 37].

PD-1 is a trans-membrane protein belonging to the CD28 family, and expresses on various immune cells especially activated and exhausted T cells [38]. Nonetheless, when and what determines PD-1 to mediate T cell activation, exhaustion or apoptosis is still a research focus. Multiple cancer cells express high level of PD-1 ligand (PD-L1) to evade T cell clearance through the PD-1/PD-L1 pathway [21]. Nowadays, various studies have highlighted the regulatory mechanisms of PD-(L)1 expression at the transcriptional, post-transcriptional, and extracellular protein levels in biopsy or surgery tumor samples [39]. However, researches focusing on more available peripheral blood remained limited. An early study showed that the main circulating lymphocyte subsets had regional differences around the world [40]. Till now, the consensus expression levels of PD-1 on circulating lymphocytes have not been established in cancer patients. Considering variability across the region, race, lifestyle, environment and tumor distribution, it is necessary to set out the reference range of the percentage of PD-1 positive cells in Chinese cancer patients, and explore the potential significance.

We observed the significantly lower percentage of PD-1 positive cells in cancer patients, which might support decreased frequency of T cell activation. Besides, in the case of acute initial immune stimulus, PD-1 is transiently highly expressed on CD4^+^ and CD8^+^ T cells. But chronic antigen exposure in tumor patients might occur more frequently impaired response to stimulus [41]. Recent study supported that the differentiation status of peripheral blood PD-1^+^ CD8^+^ cells might benefit the selection of response individuals to PD-1 inhibitors [42]. More researches are needed to explore the circulating PD-1 positive immune cells.

### Variation of immune cells accompanying with age and gender

Previous studies reported age-related markedly reduced T lymphocytes and impaired B cell mediated antibody response [10, 43, 44], but innate immunity was considered to preserve stable [45]. Although controversial, most previous studies reported an increased NK cells during aging in healthy people, but the function of NK cells was impaired [46].

In this study, cell counts of CD3^+^ T, CD3^+^CD4^+^ Th, CD3^+^CD8^+^ CTL, CD19^+^ B and the percentage of CD3^+^CD45^+^ cells, CD3^+^CD4^+^ Th cells, CD3^+^CD8^+^ CTL, CD19^+^ B cells were presented decline with advancing age. This trend was similar in both cancer and non-cancer population. More and more evidence noted that a reduction of the absolute lymphocyte count in peripheral blood correlated with a worse response and prognosis to immune checkpoint inhibitor therapy [47, 48].

However, both CD16^+^CD56^+^ NK cell counts and its percentage indicated similar increased trend with ageing in our results. The correlation between a higher baseline level of NK cell count in peripheral blood and better ICI treatment prognosis was controversial [49, 50]. Recent studies provided evidence that diverse ages of NK cells presented different functions, aged NK cells might produce more Interferon (IFN)-γ and have a robust response to infections [51]. More evidences were required to clarify the interactions, mechanisms and therapeutic prospects.

Early studies supported that age-related decline occurred less in CD3^+^CD4^+^ Th cells than CD3^+^CD8^+^ CTL, leading to an increase ratio of CD4^+^/CD8^+^ T cells in elder healthy individuals [52]. However, cancer patients exhibited different results in this study, the ratio of CD4^+^/CD8^+^ T cells kept balance with age growing. More researches mainly focused on the interaction of CD8^+^ T lymphocytes and tumor microenvironment, and ignored the important role of CD4^+^ Th cells in coordinating the innate and adaptive immunity [53]. Specific CD4^+^ T cell populations also mediated tumor killing and regression [54]. Some approved CAR-T cell products defined a higher ratio of CD4^+^/CD8^+^ T cells to predict therapeutic response in multiple myeloma [54, 55]. In cancer patients, the balanced ratio of CD4^+^/CD8^+^ T cells throughout life might be another indicator for impaired capacity of immune response.

Previous reports demonstrated the gender-related difference in lymphocyte subsets, sex associated reduction on NK and B cells in mouse model and various healthy populations [56–60]. Moreover, males were more possible to have an imbalanced ratio of CD4^+^/CD8^+^ T cells than females [61]. In our study, gender did not seem to influence the cell counts of CD45^+^, CD3^+^ T, CD3^+^CD4^+^ Th, CD3^+^CD8^+^ CTL, the percentage of CD3^+^CD45^+^ cells and CD3^+^CD8^+^ CTL in cancer patients.

By comparison, we found that females had fewer CD16^+^CD56^+^ NK cell counts and percentage, more CD19^+^ B cell counts and percentage, and higher ratio of CD4^+^/CD8^+^ T cells than males. Consistent with early studies in the same aged population, males were more possible to have an imbalanced ratio of CD4^+^/ CD8^+^ T cells than females in cancer patients. The reasons for these differences remained unclear. Neuroendocrine mechanisms including sex and stress hormones might play a role in this gender related differences [62, 63].

### Tunor stage associated immune fluctuation and age, gender influenced tumor distribution

Immune cells played double-edged role in cancer, including anti-tumorigenic and pro-tumorigenic [64]. In the current study, cell counts of CD45^+^, CD3^+^ T, CD3^+^CD4^+^ Th, CD3^+^CD8^+^ CTL, CD16^+^CD56^+^ NK and CD19^+^ B sharply decreased not only with the cancer diagnosis, but also with the tumor moving toward advanced stage. During tumor progression, cancer cells recruited a variety of immune cells to construct immunosuppressive tumor microenvironment to escape from immune attack [65]. Taken together, the interaction between the tumor cells and the host immune cells, determined the fate of disease progression or recovery.

Approximately 50% of all cancers occur in patients over 65 years old, with an estimate incidence rising to 70% as life expectancy continues to extend [47]. In this study, 599 (45.5%) cases of major 14 cancer types distributed in elder subgroup. In addition, the incidence rate of tumor in men was higher than in women. Although the age and gender influenced trend on different cancer types could be traced, the 1375 sample size in our study was far from sufficient to observe and analyze the epidemiology of tumors.

### PD-1 expression on T cells and its discrepancy in cancer patients

T cell activation could increase the percentage of PD-1^+^ T cells [66]. Early studies exhibited that PD-1 expression on T cells increased with age in preclinical studies [67, 68]. Publications also described the correlation between elevation percentage of PD-1^+^CD3^+^CD8^+^ T cells and advanced tumor stage [69]. In our data, gender did not affect the percentage of PD-1^+^CD45^+^ cells, PD-1^+^CD3^+^ T cells, PD-1^+^CD3^+^CD4^+^ Th cells and PD-1^+^CD3^+^CD8^+^ CTL. However, an increasing trend of PD-1 positive cells was presented with age and tumor stage in whole cancer patients. Inconsistent results appeared in the percentage of PD-1^+^CD3^+^CD8^+^ CTL, which increased with age but had no difference among different tumor stage. More details were needed to distinguish inflammatory tumors and further studies were required to validate in the same tumor type.

Moreover, our study showed the percentage of PD-1^+^CD45^+^ cells, PD-1^+^CD3^+^ T cells, PD-1^+^CD3^+^CD4^+^ Th cells, PD-1^+^CD3^+^CD8^+^ CTL varied among major cancer types. Head and neck, pancreatic, gynaecological, lung, esophagus and gastric cancer demonstrated a moderate to higher percentage of PD-1 positive cells than melanoma, prostate and breast cancer. These results consisted with early analysis of tumor mutational burden (TMB) in 24 cancer types, which showed higher TMB in lung, uterus, stomach, head and neck cancer, and lower TMB in kidney, prostate and breast cancer [70].

TMB was defined as the number of somatic mutations per megabase measured by panel-based or whole-exome sequencing (WES) approaches [70]. Collectively, TMB could generate tumor specific neoantigens, prime T cell activation, and initiate anti-cancer immunity, which has become an important biomarker to predict ICIs treatment response [71–73]. Considering broader WES strategy was impractical in clinical work to accurately analyse TMB [70], we analysed the expression of PD-1 on immune cells to address the regulatory mechanism of T cell priming and activation. Inconsistent result existed in melanoma, which showed high TMB in early study but low percentage of PD-1^+^ T cells in our results. The possible mechanism might be that major cutaneous melanoma in Caucasians had higher TMB, while acral and mucosal melanomas with low TMB were more prevalent in China [74]. On the whole, the expression of PD-1 on T cells might be a potential promising predictor for anti-PD-1 therapy.

Based on previous and our data, ageing-associated inflammation originated from the chronic infection or accumulation of senescent cells, resulting in up-regulation the percentage of PD-1^+^ T cells. Also, the aged immune system was responsible for tumor initiation and progression, and the hallmarks of T cell ageing manifested by the induction and expression of PD-1. Additionally, the communication among inflammation, tumorigenesis, and immunity agents was bi-directional [37]. Ultimately, T cells became exhausted and interacted with increased expression of PD-L1 on cancer cells, and inhibited anti-cancer activity. Recent results revealed that PD-L1 expression played a pivotal role in the age-related accumulation of senescent cells, thus, the elimination of T cell exhaustion by anti-PD-1 treatment maybe a potential strategy to improve anti-ageing regimen, more than anti-tumor activity [75].

## Conclusion

As ageing, chronic inflammation and senescence cells accumulation co-promoting, cancer incidence increased and the tumor immunity cycle drived simultaneously. In this study, we used flow cytometric to evaluate a series of immune changes in pan-cancer patients and healthy control. Cell counts of CD3^+^ T, CD3^+^CD4^+^ Th, CD3^+^CD8^+^ CTL, CD16^+^CD56^+^ NK, CD19^+^ B and the percentage of PD-1 positive cells decreased in cancer patients, supported impaired innate and adaptive immunity and lower T cell activation. For cancer patients, cell counts of CD3^+^ T, CD3^+^CD4^+^ Th, CD3^+^CD8^+^ CTL, CD19^+^ B decreased with advancing age and stage. However, CD16^+^CD56^+^ NK cell counts increased with age, male hormones, but decreased with tumor stage. Moreover, the percentage of PD-1 positive cells elevated with ageing and tumor progression, and affected by cancer type. Further in-depth comparison between the percentage of PD-1 positive cells in our data and the TMB reported in previous studies suggested that, the percentage of PD-1 positive cells might be a potential promising biomarker for ICI treatment.

### Methods

#### Study cohort and ethics

Between Jan 2018 and July 2021, a total of 1375 cancer patients at the Affiliated Cancer Hospital of Zhengzhou University & Henan Cancer Hospital and 275 age and gender balanced healthy volunteers were recruited in this study (Supplementatry Fig. 6). There were 770 males (56%) and 605 females (44%) in cancer patient group, and the median age was 58 years old (range 18–89). The matched details of age and gender were shown in Supplementary Fig. 1. Subjects testing positive for HIV, acute active infection and connective tissue disease and experienced cancer therapies were excluded. The subjects were classified as young adults (18–35), middle-aged (36–60) and elderly (61–90). This study was approved by the Ethical Committee of Henan Cancer Hospital.

### Flow cytometry

Peripheral whole blood samples were collected into EDTA-vacutainers and used directly for flow cytometry staining. Each sample was labeled as “T-B-NK cells”, “PD-1-FMO control” and “PD-1” separately. For each tube, appropriate amounts of fluorochrome-conjugated monoclonal antibody reagents were added to 100µL whole blood sample in sequence, and then vortexed gently. Cells were stained with the following surface antibodies to determine the T cell lineage (T-B-NK tube): anti-CD45 PerCP-Cy™5.5 (BD bioscience, Cat.No.652,803), anti-CD3 PE/Cyanine7 (Biolegend, Cat.No. 344,816), anti-CD4 PE (Biolegend, Cat.No.317,410), anti-CD8a FITC (Biolegend, Cat.No.301,050), anti-CD19 APC (Biolegend, Cat.No.302,212), anti-CD16 PE (Biolegend, Cat.No.360,704), anti-CD56 PE (Biolegend, Cat.No.362,508). For PD-1 positive T cells, in addition to the above mentioned anti-CD45, anti-CD3, anti-CD4, anti-CD8 antibodies, anti-CD279 (PD-1) APC (Biolegend, Cat.No.329,908) was used (PD-1 tube). APC anti-human IgG1, κ Isotype Ctrl Antibody (Biolegend, Cat.No.400,120) was used for isotype control of PD-1 (PD-1-FMO). Surface markers were acquired by FACS CantoII (BD Biosciences) within 2 h after stained. The data was analyzed with FlowJo software (Tree Star, Inc.).

### Cancer type

Flow cytometry was used to analyze a spectrum of different tumor types including lung cancer (non-small and small cell lung cancer, *n* = 307), esophagus and gastric cancer (*n* = 212), melanoma (*n* = 166), colorectal carcinoma (*n* = 129), kidney cancer (*n* = 121), gynecological cancer (cervical, ovarian and uterine cancer, *n* = 94), hepatobiliary cancer (*n* = 84), breast cancer (*n* = 66), head and neck cancer (*n* = 36), bone and soft tissue sarcoma (*n* = 35), bladder cancer (*n* = 21), pancreatic cancer (*n* = 18), lymphoma (*n* = 14), prostate cancer (*n* = 13), occult primary cancer (*n* = 13), neuroendocrine and adrenal tumor (*n* = 9), as well as other types of cancer (thyroid, thymus, mesothelioma, leukemia, penis, small intestine, central nervous system, gastrointestinal stromal tumor, *n* = 37).

### Statistical analysis

Categorical variables were compared using the Chi-squared test. Fisher’s exact tests were used to analyze the differences in clinical demographics, characteristics, and balances among the groups. As for data containing more than two groups, they were compared by one-way ANOVA test followed by the Turkey test. The reference range of the percentage of PD-1 positive cells were demonstrated by median + 95% confidential interval (CI) and compared by Mann-Whitney test between groups. The Pearson correlation coefficient was computed to evaluate correlations between two parameters. SPSS 17.0 software and GraphPad Prism 9 were used for statistical analysis and graphs. *P* value < 0.05 was considered significant.

### Limitation

Firstly, human immunity is a complex system and multi-level regulated process. Whereas, our study explored peripheral immune cell types to analyse and understand the whole immunity of the individuals, and did not have comparison with immune organs and mechanism investigations. Secondly, the patients were recruited from a single cancer center, so tumor heterogeneity among different regions and epidemiology was inevitable. Thirdly, further investigations are needed to compare with tumor microenvironment and clarify the correlation with prognosis to better understand the value of circulating PD-1 positive cells.

### Electronic supplementary material

Below is the link to the electronic supplementary material.

**Supplementary Material 1: Supplementary Fig. 1.** The age and gender constituents in cancer patients and healthy control (A) The case constituents of age subgroups in cancer patients and healthy control group. (The case number details are shown in the supplementary Table e1). (B) The cases constituent of gender in cancer patients and healthy control group. Categorical variables were compared using the Chi-squared test. Fisher’s exact tests were used to analyse the demographics among groups

**Supplementary Material 2: Supplementary Fig. 2.** The cell counts and percentages were not affected by gender in cancer patients. (A-D) The absolute number of CD45^+^ cells, CD3^+^ T cells, CD3^+^CD4^+^ Th cells, CD3^+^CD8^+^ CTL in male and female cancer patients. (E-F) The percentage of CD3^+^CD45^+^ cells, and CD3^+^CD8^+^ CTL in male and female cancer patients. Data were shown as mean ± SEM. Statistic differences between two groups were detected by unpaired t-tests

**Supplementary Material 3: Supplementary Fig. 3.** The cell percentage was not affected by stage in cancer patients (A-E) The percentage of CD3^+^CD45^+^ cells, CD16^+^ CD56^+^ NK cells, CD19^+^ B cells, CD3^+^CD4^+^ Th cells, CD3^+^CD8^+^ CTL among early, middle and late stage. (F) The ratio of CD4^+^/CD8^+^ T cells in early, middle and late stage of cancer patients. Data were shown as mean ± SEM and analyzed by one-way ANOVA test followed by the Turkey test

**Supplementary Material 4: Supplementary Fig. 4.** The comparison of immune cell counts among major cancer types The absolute number of CD45^+^ cells, CD3^+^ T cells, CD3^+^CD4^+^ Th cells, CD3^+^CD8^+^ CTL, CD19^+^ B cells, CD16^+^ CD56^+^ NK cells in cancer subtypes. Data were shown as mean ± SEM and analyzed by one-way ANOVA test followed by the Turkey test

**Supplementary Material 5: Supplementary Fig. 5.** The percentage of PD-1 positive cells and its variation in healthy control. (A-D) The correlation between the percentage of PD-1^+^CD45^+^ cells, PD-1^+^CD3^+^ T cells, PD-1^+^CD3^+^CD4^+^ Th cells, PD-1^+^CD3^+^CD8^+^ CTL and age in healthy control (*n* = 275). The Pearson correlation coefficient was computed to evaluate correlations between two parameters. (E-H) The percentage of PD-1^+^CD45^+^ cells, PD-1^+^CD3^+^ T cells, PD-1^+^CD3^+^CD4^+^ Th cells, and PD-1^+^CD3^+^CD8^+^ CTL in male (*n* = 150) and female (*n* = 125) healthy populations. Data were shown as mean ± SEM. Statistic differences between two groups were detected by unpaired t-tests

**Supplementary Material 6**

**Supplementary Material 7**

**Supplementary Material 8**

**Supplementary Material 9**

## Data Availability

All data generated or analysed during this study are included in this published article and its supplementary information files.

## Abbreviations

PD-1: Programmed cell death protein-1
CTL: Cytotoxic T Lymphocyte
TME: Tumor microenvironment
NSCLC: Non-small cell lung cancer
PFS: Progression free survival
PBMCs: Peripheral blood mononuclear cells
HSCs: Hematopoietic stem cells
HPV: Human papillomavirus
EBV: Epstein-Barr virus
HBV: Hepatitis B virus
TMB: Tumor mutational burden
WES: Whole-exome sequencing
