# Supplementary figures and images for: The inter-link of ageing, cancer and immunity: findings from real-world retrospective study
Source: Immun Ageing. 2023 Dec 15;20:75. doi: 10.1186/s12979-023-00399-9 (PMC10722682; doi:10.1186/s12979-023-00399-9)

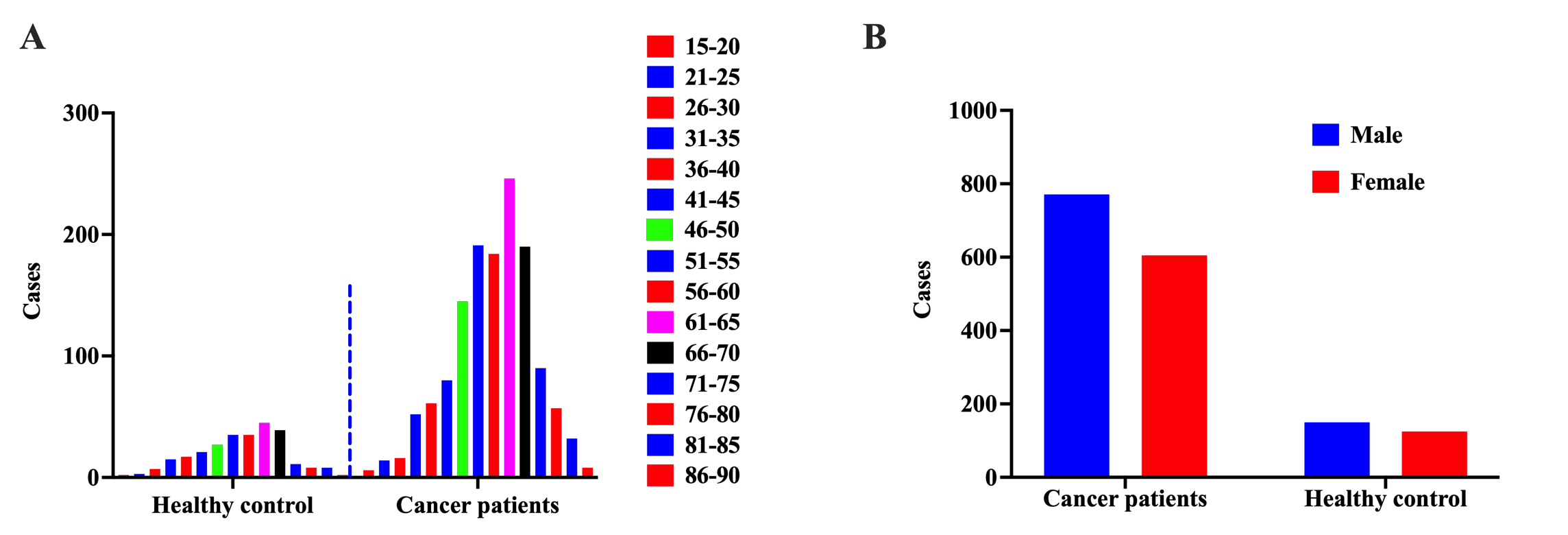

Supplement: Supplementary file 1 — Supplementary Material 1: Supplementary Fig. 1. The age and gender constituents in cancer patients and healthy control (A) The case constituents of age subgroups in cancer patients and healthy control group. (The case number details are shown in the supplementary Table e1). (B) The cases constituent of gender in cancer patients and healthy control group. Categorical variables were compared using the Chi-squared test. Fisher’s exact tests were used to analyse the demographics among groups [file 12979_2023_399_MOESM1_ESM.tiff]

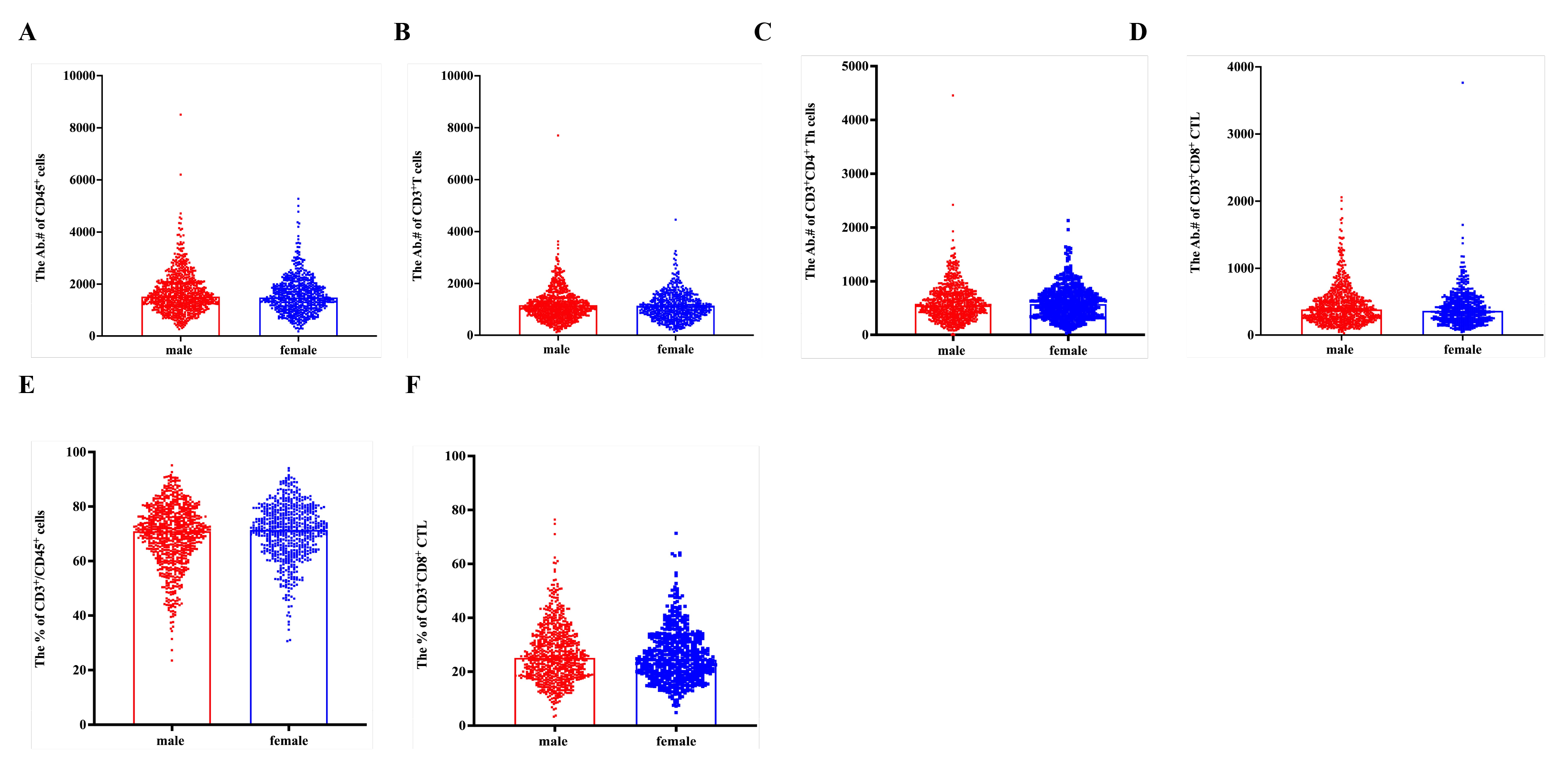

Supplement: Supplementary file 2 — Supplementary Material 2: Supplementary Fig. 2. The cell counts and percentages were not affected by gender in cancer patients. (A-D) The absolute number of CD45+ cells, CD3+ T cells, CD3+CD4+ Th cells, CD3+CD8+ CTL in male and female cancer patients. (E-F) The percentage of CD3+CD45+ cells, and CD3+CD8+ CTL in male and female cancer patients. Data were shown as mean ± SEM. Statistic differences between two groups were detected by unpaired t-tests [file 12979_2023_399_MOESM2_ESM.tif]

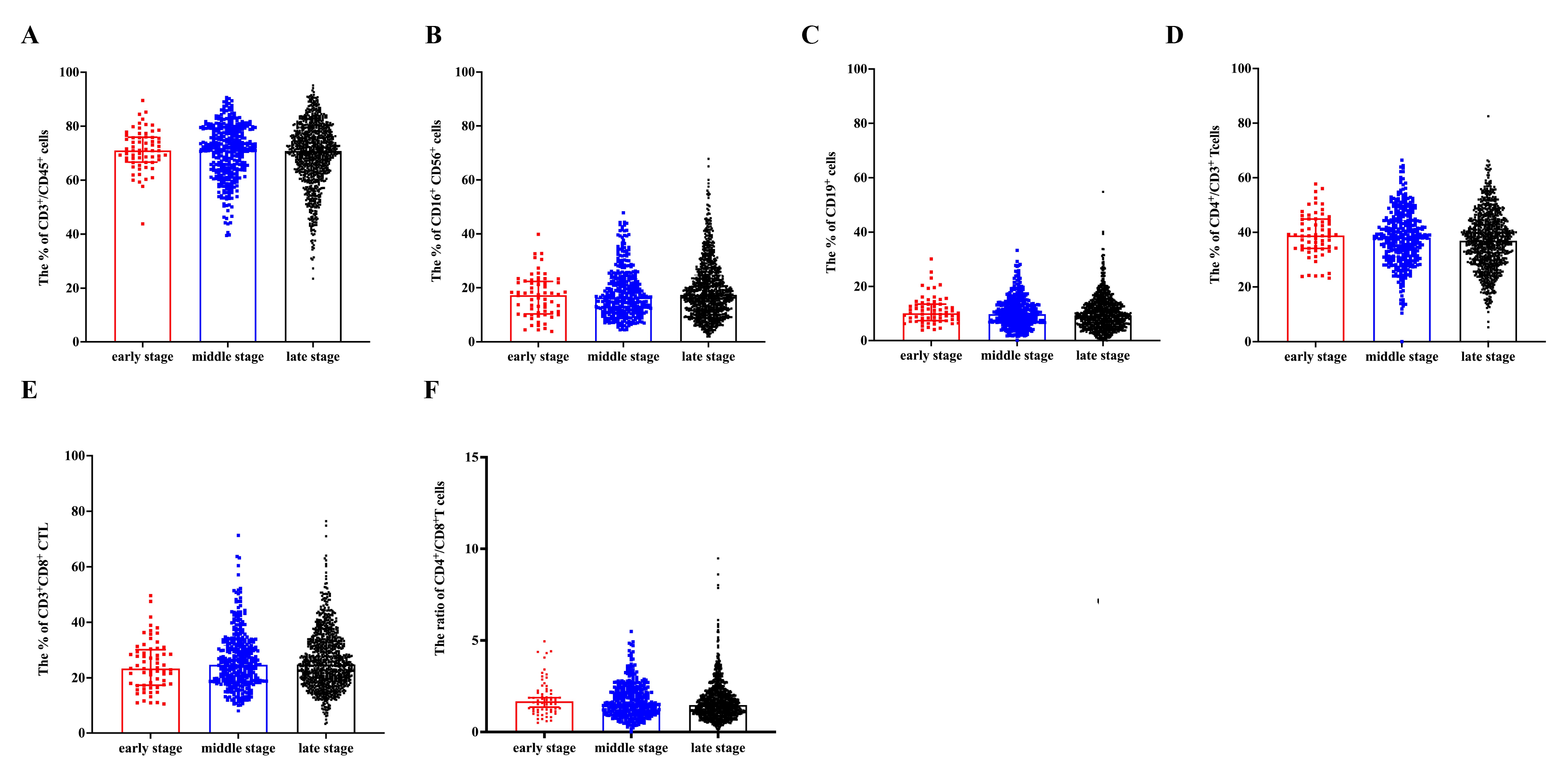

Supplement: Supplementary file 3 — Supplementary Material 3: Supplementary Fig. 3. The cell percentage was not affected by stage in cancer patients (A-E) The percentage of CD3+CD45+ cells, CD16+ CD56+ NK cells, CD19+ B cells, CD3+CD4+ Th cells, CD3+CD8+ CTL among early, middle and late stage. (F) The ratio of CD4+/CD8+ T cells in early, middle and late stage of cancer patients. Data were shown as mean ± SEM and analyzed by one-way ANOVA test followed by the Turkey test [file 12979_2023_399_MOESM3_ESM.tif]

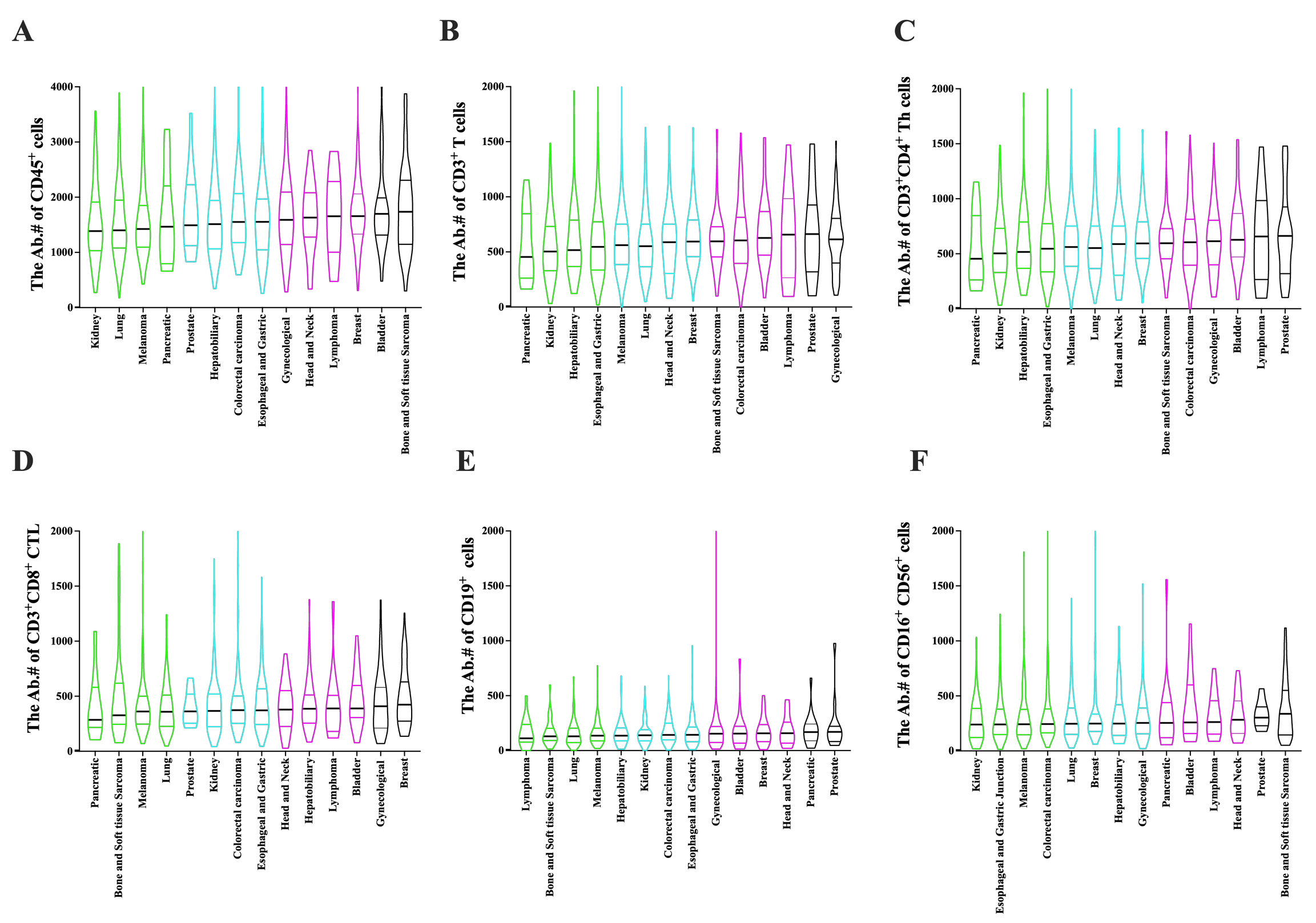

Supplement: Supplementary file 4 — Supplementary Material 4: Supplementary Fig. 4. The comparison of immune cell counts among major cancer types The absolute number of CD45+ cells, CD3+ T cells, CD3+CD4+ Th cells, CD3+CD8+ CTL, CD19+ B cells, CD16+ CD56+ NK cells in cancer subtypes. Data were shown as mean ± SEM and analyzed by one-way ANOVA test followed by the Turkey test [file 12979_2023_399_MOESM4_ESM.tiff]

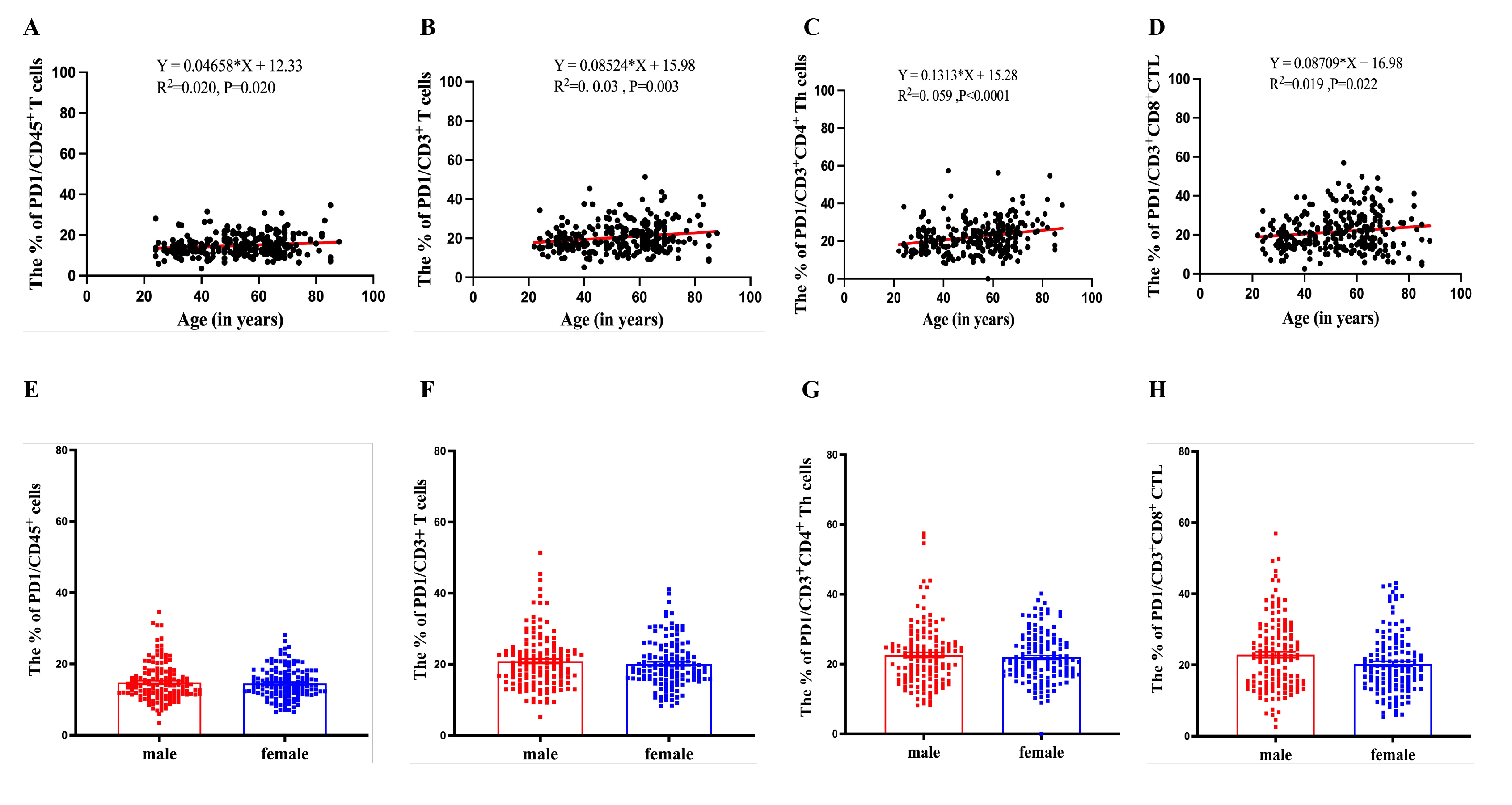

Supplement: Supplementary file 5 — Supplementary Material 5: Supplementary Fig. 5. The percentage of PD-1 positive cells and its variation in healthy control. (A-D) The correlation between the percentage of PD-1+CD45+ cells, PD-1+CD3+ T cells, PD-1+CD3+CD4+ Th cells, PD-1+CD3+CD8+ CTL and age in healthy control (n = 275). The Pearson correlation coefficient was computed to evaluate correlations between two parameters. (E-H) The percentage of PD-1+CD45+ cells, PD-1+CD3+ T cells, PD-1+CD3+CD4+ Th cells, and PD-1+CD3+CD8+ CTL in male (n = 150) and female (n = 125) healthy populations. Data were shown as mean ± SEM. Statistic differences between two groups were detected by unpaired t-tests [file 12979_2023_399_MOESM5_ESM.tif]

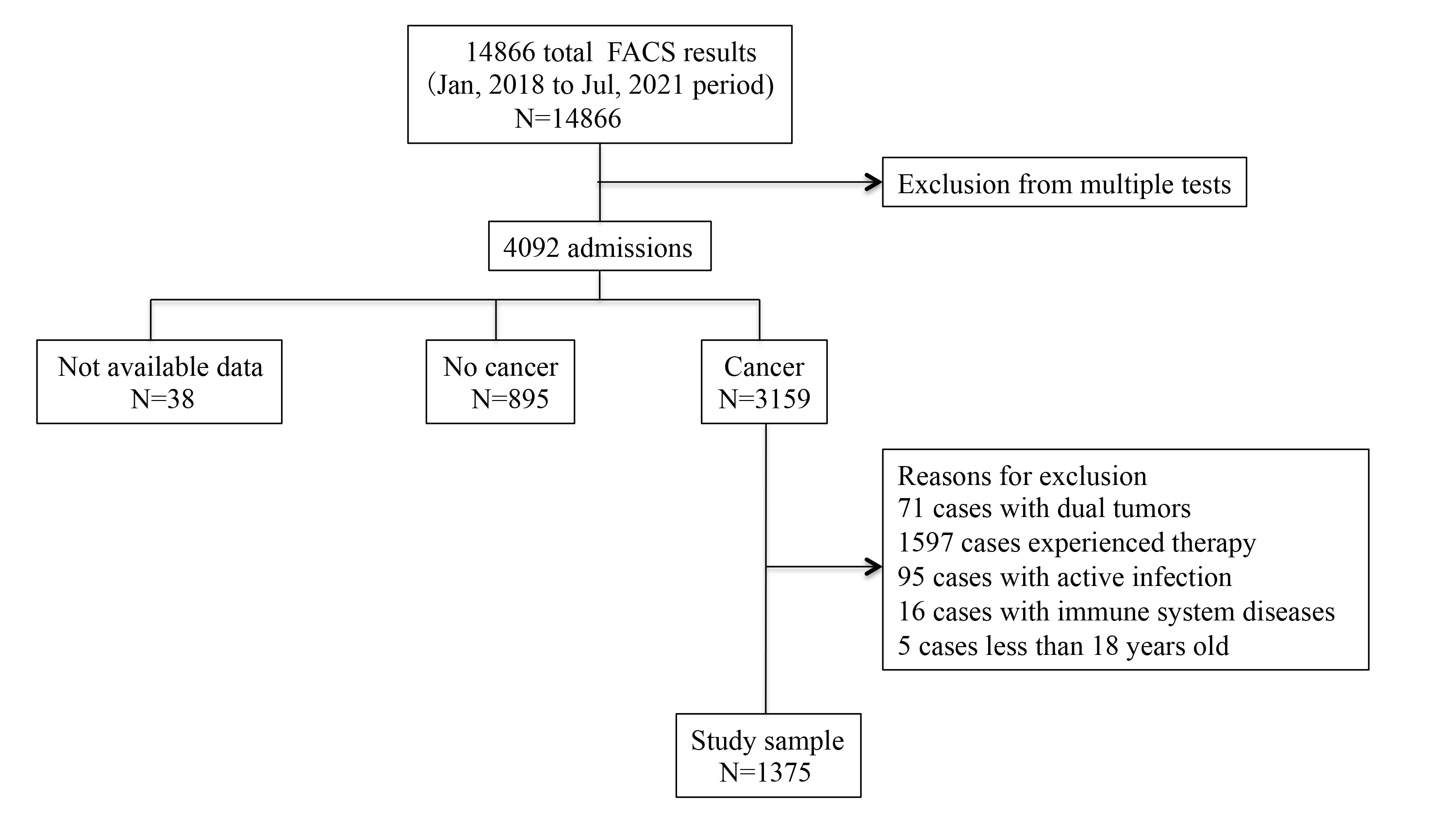

Supplement: Supplementary file 6 — Supplementary Material 6 [file 12979_2023_399_MOESM6_ESM.tif]
