# Supplementary material for: The inter-link of ageing, cancer and immunity: findings from real-world retrospective study
Source: Immun Ageing. 2023 Dec 15;20:75. doi: 10.1186/s12979-023-00399-9 (PMC10722682; doi:10.1186/s12979-023-00399-9)
Supplement: Supplementary file 7 — Supplementary Material 7 [file 12979_2023_399_MOESM7_ESM.docx]

**Supplementary Table 1 Comparison of immune cell counts in peripheral blood between cancer patients and healthy control**

|  | Cancer patients  (median 95% CI) | Cancer patients  (mean 95% CI) | Healthy control  (mean 95% CI) | P value |
| --- | --- | --- | --- | --- |
| CD45^+^ cells | 1496 (1447-1550) | 1622 (1581-1662) | 2615 (1530-3700) | p<0.0001 |
| CD3^+^ T cells | 1053 (1024-1085) | 1136 (1106-11666) | 1815 (770-2860) | p<0.0001 |
| CD3^+^CD4^+^ Th cells | 557.4 (542.4-582.5) | 602.2 (585-619.4) | 927 (414-1440) | p<0.0001 |
| CD3^+^CD8^+^ CTL cells | 370.9 (356.9-387.7) | 426 (411.3-440.7) | 744 (238-1250) | p<0.0001 |
| CD16^+^CD56^+^ NK cells | 251.1 (238.9-261.5) | 308.7 (296.1-321.4) | 630 (150-1110) | p<0.0001 |
| CD19^+^ B cells | 140.4 (134.3- 147) | 167 (296.1-321.4) | 325 (90-560) | p<0.0001 |

The absolute number of CD45^+^ cells, CD3^+^ T cells, CD3^+^CD4^+^ Th cells, CD3^+^CD8^+^ CTL cells, CD16^+^CD56^+^ NK cells, CD19^+^ B cells in cancer patients (n=1375) were detected by flow cytometry. Data were shown as median+95% CI and mean+95% CI separately. The absolute number of the above lymphocyte subtypes in the healthy control group were acquired from BD Bioscience Company and demonstrated as mean+95% CI. Data were analysed by Mann–Whitney test between groups. p values were indicated in the table.
