# Supplementary material for: The inter-link of ageing, cancer and immunity: findings from real-world retrospective study
Source: Immun Ageing. 2023 Dec 15;20:75. doi: 10.1186/s12979-023-00399-9 (PMC10722682; doi:10.1186/s12979-023-00399-9)
Supplement: Supplementary file 8 — Supplementary Material 8 [file 12979_2023_399_MOESM8_ESM.docx]

Supplementary Table 2 Distribution of cancer types in age and gender subgroups

| Cancer type and distribution | Age | | | Total | χ^2^ value | P value |
| --- | --- | --- | --- | --- | --- | --- |
|  | ≤35 | 36-60 | > 60 |  |  |  |
| Breast  Male  Female | 7  0  7 | 41  0  41 | 18  0  18 | 66 | － | － |
| Prostate  Male  Female | 0  0  0 | 1  1  0 | 12  12  0 | 13 | － | － |
| Melanoma  Male  Female | 19  7  12 | 84  37  47 | 63  28  35 | 166 | 0.375 | 0.829 |
| Lymphoma  Male  Female | 2  2  0 | 3  2  1 | 9  4  5 | 14 | 2.204 | 0.332 |
| Bone and soft tissue Sarcoma  Male  Female | 12  10  2 | 17  7  10 | 6  5  1 | 35 | 6.655 | 0.036 |
| Kidney  Male  Female | 8  7  1 | 73  47  26 | 40  29  11 | 121 | 2.211 | 0.331 |
| Colorectal carcinoma  Male  Female | 7  5  2 | 69  37  32 | 53  34  19 | 129 | 1.851 | 0.396 |
| Lung  Male  Female | 10  7  3 | 128  78  50 | 169  107  62 | 307 | 0.421 | 0.810 |
| Bladder  Male  Female | 1  1  0 | 6  4  2 | 14  10  4 | 21 | 0.467 | 0.792 |
| Hepatobiliary  Male  Female | 3  2  1 | 49  41  8 | 32  21  11 | 84 | 3.632 | 0.163 |
| Esophageal and Gastric  Male  Female | 5  4  1 | 84  62  22 | 123  88  35 | 212 | 0.268 | 0.874 |
| Gynecological  Male  Female | 6  0  6 | 54  0  54 | 34  0  34 | 94 | － | － |
| Pancreatic  Male  Female | 0  0  0 | 9  5  4 | 9  5  4 | 18 | 0.000 | >0.999 |
| Head and neck  Male  Female | 0  0  0 | 19  15  4 | 17  13  4 | 36 | 0.032 | 0.858 |
| Total  Male  Female | 80  45  35 | 637  336  301 | 599  356  243 | 1316  737  579 | 5.601 | 0.061 |

The cancer type distribution was shown in the table. The cancer patients were divided into three groups according to age (≤35, 36-60, >60 years old). The gender and age constituents of each cancer type were shown in the table. Data were shown as case numbers and percentages of the subgroups. Categorical variables were compared using the Chi-squared test. Fisher’s exact tests were used to analyse the demographics among the groups. χ2 value and P value were indicated in the table.
