# Supplementary material for: The inter-link of ageing, cancer and immunity: findings from real-world retrospective study
Source: Immun Ageing. 2023 Dec 15;20:75. doi: 10.1186/s12979-023-00399-9 (PMC10722682; doi:10.1186/s12979-023-00399-9)
Supplement: Supplementary file 9 — Supplementary Material 9 [file 12979_2023_399_MOESM9_ESM.docx]

S Table Characteristics of age and gender matching details

|  | Cancer patients (n) | Healthy control (n) | χ2 value | P value |
| --- | --- | --- | --- | --- |
| Total | 1375 | 275 |  |  |
| Male | 770 (56.0%) | 150(54.5%) | 0.197 | 0.658 |
| Female | 605(44.0%) | 125(45.5%) |  |  |
| Age (18~25) | 30 | 5 |  |  |
| Male | 12(60.0%) | 3(60.0%) | 0.000 | 1.000 |
| Female | 8(40.0%) | 2(40.0%) |  |  |
| Age (26~30) | 16 | 7 |  |  |
| Male | 12(75.0%) | 5(71.4%) | 0.031 | 0.861 |
| Female | 4(25.0%) | 2(28.6%) |  |  |
| Age (31~35) | 52 | 15 |  |  |
| Male | 24(46.2%) | 7(46.7%) | 0.001 | 0.972 |
| Female | 28(53.8%) | 8(53.3%) |  |  |
| Age (36~40) | 62 | 17 |  |  |
| Male | 30(48.4%) | 8(47.1%) | 0.009 | 0.923 |
| Female | 32(51.6%) | 9(52.9%) |  |  |
| Age (41~45) | 80 | 21 |  |  |
| Male | 38(47.5%) | 10(47.6%) | 0.000 | 0.992 |
| Female | 42(52.5%) | 11(52.4%) |  |  |
| Age (46~50) | 145 | 27 |  |  |
| Male | 76(52.4%) | 15(55.6%) | 0.090 | 0.764 |
| Female | 69(47.6%) | 12(44.4%) |  |  |
| Age (51~55) | 191 | 35 |  |  |
| Male | 100(52.4%) | 19(54.3%) | 0.044 | 0.834 |
| Female | 91(47.6%) | 16(45.7%) |  |  |
| Age (56~60) | 185 | 35 |  |  |
| Male | 108(58.4%) | 21(60.0%) | 0.032 | 0.858 |
| Female | 77(41.6%) | 14(40.0%) |  |  |
| Age (61~65) | 246 | 46 |  |  |
| Male | 139(56.5%) | 24(52.2%) | 0.295 | 0.587 |
| Female | 107(52.2%) | 22(47.8%) |  |  |
| Age (66~70) | 190 | 38 |  |  |
| Male | 116(61.1%) | 21(55.3%) | 0.443 | 0.506 |
| Female | 74(38.9%) | 17(44.7%) |  |  |
| Age (71~75) | 90 | 11 |  |  |
| Male | 53(58.9%) | 6(54.4%) | 0.076 | 0.783 |
| Female | 37(41.4%) | 5(45.5%) |  |  |
| Age (76~80) | 58 | 8 |  |  |
| Male | 36(62.1%) | 5(62.5%) | 0.001 | 0.981 |
| Female | 22(37.9%) | 3(37.5%) |  |  |
| Age (81~85) | 32 | 8 |  |  |
| Male | 22(68.8%) | 5(62.5%) | 0.000 | 1.000 |
| Female | 10(31.3%) | 3(37.5%) |  |  |
| Age (86~90) | 8 | 2 |  |  |
| Male | 4(50.0%) | 1(50.0%) | 0.000 | 1.000 |
| Female | 4(50.0%) | 1(50.0%) |  |  |

The age and gender constituents of cancer patients (n=1375) and healthy control (n=275) were shown in the table. Data were shown as case numbers and percentages of the subgroups. Categorical variables were compared using the Chi-squared test. Fisher’s exact tests were used to analyse the demographics among the groups. χ2 value and P value were indicated in the table.
